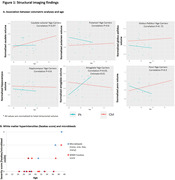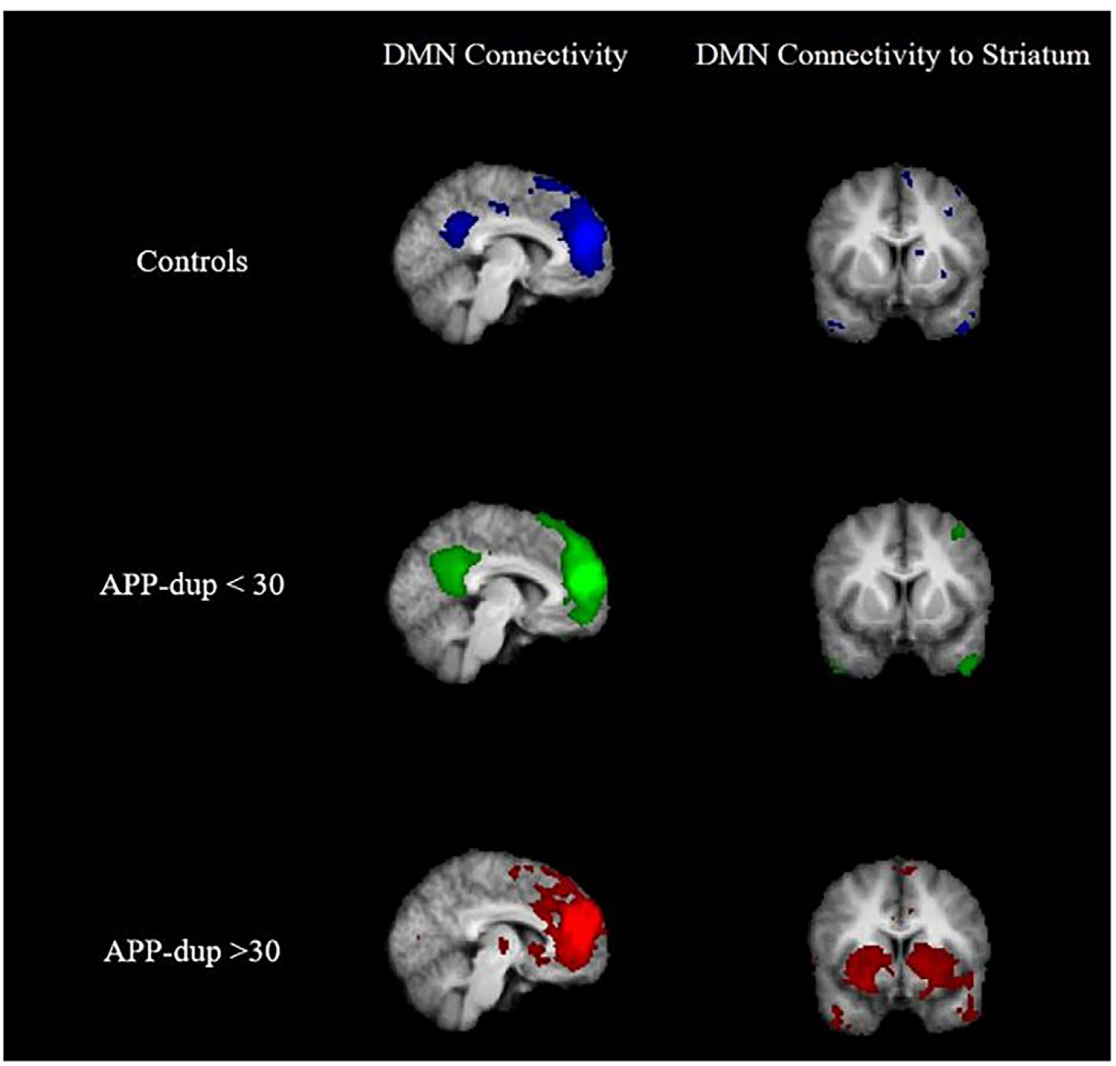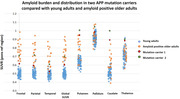# Early basal ganglia imaging abnormalities in pre‐symptomatic and early symptomatic novel APP duplication carriers

**DOI:** 10.1002/alz.095158

**Published:** 2025-01-09

**Authors:** Rotem Paz, Eyal Bergmann, Ayelet Eran, Yael Rozen, Sapir Golan, Goni Merhav, Chen Hoffmann, Liran Domachevsky, Michal S Beeri, Limor Kalfon, Tzipora Falik‐Zaccai, Judith Aharon‐Peretz, Itamar Kahn, Orit H. Lesman‐Segev, Ramit Ravona‐Springer

**Affiliations:** ^1^ Rappaport Faculty of Medicine, Technion, Haifa Israel; ^2^ Cognitive Neurology Institute, Rambam Health Care Campus, Haifa Israel; ^3^ Department of Psychiatry, Rambam Health Care Campus, Haifa Israel; ^4^ Department of Diagnostic Imaging, Rambam Health Care Campus, Haifa Israel; ^5^ The Joseph Sagol Neuroscience Center, Sheba Medical Center, Tel Hashomer Israel; ^6^ Faculty of Medical and Health Science, Tel Aviv University, Tel Aviv Israel; ^7^ Department of Diagnostic Imaging, Sheba Medical Center, Tel Hashomer Israel; ^8^ Institute of Human Genetics, Galilee Medical Center, Nahariya Israel; ^9^ The Azrieli Faculty of Medicine, Bar Ilan, Safed Israel; ^10^ Department of Neuroscience, Zuckerman Mind Brain Behavior Institute, Columbia University, New York USA; ^11^ Tel Aviv University, Tel Aviv Israel

## Abstract

**Background:**

We aim to describe the imaging findings in pre‐symptomatic and early‐symptomatic individuals carrying a recently described novel APP duplication rearrangement causing early‐onset AD (EOAD).

**Method:**

We studied individuals from one pedigree that carry APP duplication and CH 5 mutation gain that had structural and resting state functional (rs‐f) brain MRI. Non‐carrier family members were assessed as controls. Volumetric analysis was performed using Freesurfer and normalized to total intracranial volume. Fazekas scores for white matter hyperintensities (WMH) and microbleed count were performed visually by two neuroradiologists. rs‐fMRI seed to whole brain analysis was used to create functional connectivity maps of the default mode network (DMN). F18‐Flutemetamol amyloid‐PET was available for two of the mutation carriers. Amyloid deposition was quantified using SUVR with the pons as reference region and compared to SUVR of young adults and amyloid‐positive older adults (Ab+OA).

**Result:**

Fourteen mutation carriers (mean age 29.8[19‐39], 8 (57%) F, median education 12Y[11‐14Y], mean MMSE 28[23‐30]), and nine non‐carrier family members were included (36.8Y[19‐56Y], 5 (55%)F, 12Y[12‐19], 29[27‐30]). Volumetric analysis revealed an increase in amygdala volume (log) (estimate = 0.01, p = 0.03) and a (non‐statistically significant) trend toward decrease in the putamen, globus pallidus, and pons volume with age in mutation carriers (Figure 1A). WMHs and microbleeds were found in some mutation carriers from the age of 28Y (Figure 1B). rs‐fMRI showed a trend toward a disconnection between the anterior and the posterior component of the DMN in the APP‐dup carriers >30Y (n = 6) compared with non‐carrier (n = 8; U = 10, p = 0.08). Moreover, increased connectivity was found between the anterior component of the DMN and the striatum compared to carriers <30Y (n = 8, U = 9, p = 0.06) and to non‐carriers (U = 9, p = 0.06) (Figure 2). Cortical amyloid deposition was high but within the range of Ab+OA, and extremely high, above the level of deposition in Ab+OA in the putamen, caudate, and thalamus (Figure 3).

**Conclusion:**

We found early basal ganglia abnormalities in presymptomatic and early symptomatic novel APP duplication mutation carriers, including high amyloid deposition, DMN disconnection, increased connectivity between anterior DMN and striatum, and volumetric alterations. These findings point to the basal ganglia as a region of early pathology that requires further research.